# Rapid Improvement of Refractory Pruritus in Extragenital and Perianal Lichen Sclerosus With Nemolizumab: A Case Report

**DOI:** 10.7759/cureus.115221

**Published:** 2026-08-26

**Authors:** Corey S Frucht, Bruce D Ragsdale

**Affiliations:** 1 Dermatology, Santa Barbara Skin Care, Santa Barbara, USA; 2 Dermatopathology, Western Pathology, Inc., San Luis Obispo, USA

**Keywords:** chronic pruritus, dermatopathology, extragenital lichen sclerosus, interleukin-31, koebner phenomenon, lichen sclerosus, narrowband ultraviolet b, nemolizumab, perianal lichen sclerosus, pruritus

## Abstract

Lichen sclerosus is a chronic inflammatory dermatosis that most commonly affects anogenital skin but may also involve extragenital sites. Pruritus can be severe and may persist despite standard anti-inflammatory therapy. Nemolizumab is a monoclonal antibody targeting interleukin-31 receptor alpha, a pathway involved in chronic itch.

We report a 68-year-old woman with biopsy-proven lichen sclerosus involving the upper back, chest, shoulders, and perianal area, with several truncal lesions localizing to surgical scars from prior post-bariatric body-contouring surgery. She reported remote genital involvement several years earlier. At the eight-week follow-up, shiny white plaques involved the perianal area, but no vulvar involvement was identified. The patient had severe pruritus affecting sleep and daytime function. Prior treatments included betamethasone dipropionate 0.05% cream, intralesional triamcinolone acetonide, and office-based narrowband ultraviolet B phototherapy, the latter producing remission from 2021 until early 2026 before recurrence.

Because of recurrent severe pruritus, nemolizumab was started off-label with a 60 mg subcutaneous loading dose followed by 30 mg every four weeks. No other topical, systemic, or phototherapy treatment was used after nemolizumab initiation through the first eight weeks. At four-week follow-up, patient-reported pruritus improved from 8/10 to 0/10. At eight-week follow-up, pruritus remained markedly improved, although she reported a transient flare of burning in the perianal area several weeks earlier that had since markedly improved. No adverse effects occurred. Examination continued to show shiny white plaques, including perianal involvement, without clear morphologic remission. A short pulse of clobetasol 0.05% ointment was initiated for perianal disease at the eight-week visit while nemolizumab was continued.

This case suggests that nemolizumab may provide rapid symptomatic relief of refractory pruritus associated with lichen sclerosus, even without early morphologic resolution.

## Introduction

Lichen sclerosus is a chronic inflammatory dermatosis characterized clinically by white atrophic papules and plaques and histologically by epidermal attenuation, superficial dermal sclerosis, and a variable lymphocytic infiltrate. It most commonly affects anogenital skin, although extragenital involvement is well recognized and may involve the trunk, neck, shoulders, and proximal extremities [1-3]. Patients may be asymptomatic or may experience marked pruritus, burning, pain, dyspareunia, and functional impairment, particularly in anogenital disease [1,2].

High-potency or ultrapotent topical corticosteroids remain the cornerstone of treatment. Additional therapies that have been used in selected refractory cases include topical calcineurin inhibitors, topical and systemic retinoids, intralesional corticosteroids, phototherapy, and systemic immunomodulatory agents [1,4-6]. Extragenital disease may be especially challenging when lesions are widespread, symptomatic, or only partially responsive to topical therapy [2,3].

Chronic pruritus reflects complex interactions among keratinocytes, immune cells, and peripheral sensory neurons. Neuroimmune itch signaling may involve cytokines, neuropeptides, proteases, histamine, and other mediators that sensitize or activate pruriceptive pathways. Interleukin-31 is an important neuroimmune mediator of chronic itch. Nemolizumab is a humanized monoclonal antibody targeting interleukin-31 receptor alpha and has demonstrated antipruritic efficacy in prurigo nodularis and atopic dermatitis [7,8]. However, direct evidence implicating interleukin-31 in lichen sclerosus is limited, and the rationale for using nemolizumab in this setting is extrapolated from other pruritic inflammatory dermatoses. Data on nemolizumab for lichen sclerosus-associated pruritus remain extremely limited. We report a case of biopsy-proven truncal lichen sclerosus with later documented perianal involvement in which severe refractory pruritus improved rapidly after off-label initiation of nemolizumab, despite a lack of early morphologic improvement.

## Case presentation

A 68-year-old woman presented with biopsy-proven lichen sclerosus involving the upper back, chest, and shoulders. She reported remote genital involvement several years earlier, but at presentation the clinically documented disease was truncal. The eruption began in early 2020 and consisted of shiny white atrophic plaques involving less than 5% body surface area. Several truncal plaques were localized within or along scars from prior post-bariatric body-contouring surgery. There was no history of radiation therapy. A representative truncal lesion on the right upper back is shown in Figure 1.

**Figure 1:**
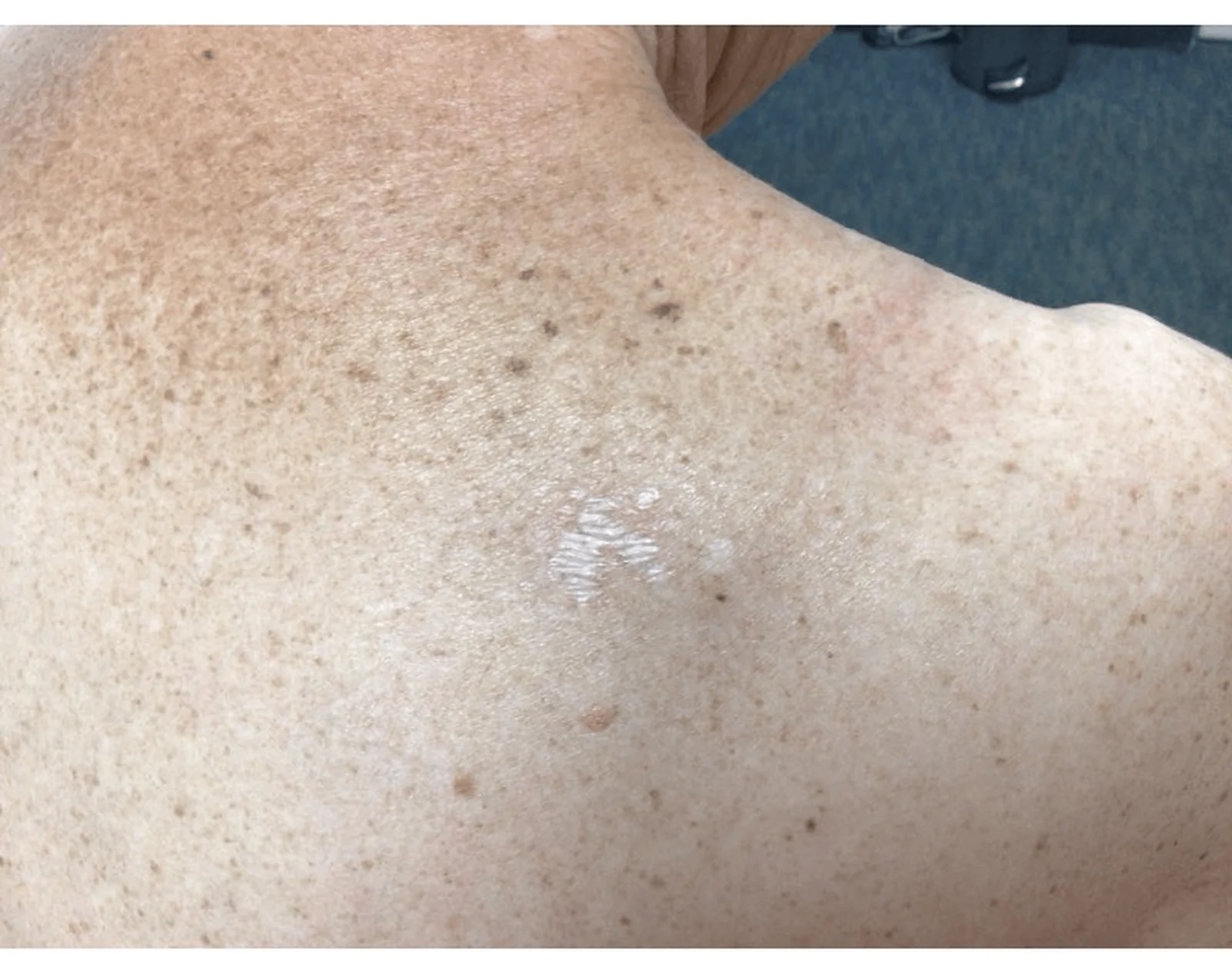
Clinical appearance Extragenital lichen sclerosus on the right upper back. Clinical photograph showing a representative shiny white atrophic plaque.

A punch biopsy of the left shoulder performed in 2020 showed lichen sclerosus. Histopathology demonstrated epidermal attenuation and superficial dermal hyalinization beneath an orthokeratotic epidermis, supporting the clinical diagnosis (Figure 2). A periodic acid-Schiff stain was negative for fungal organisms.

**Figure 2:**
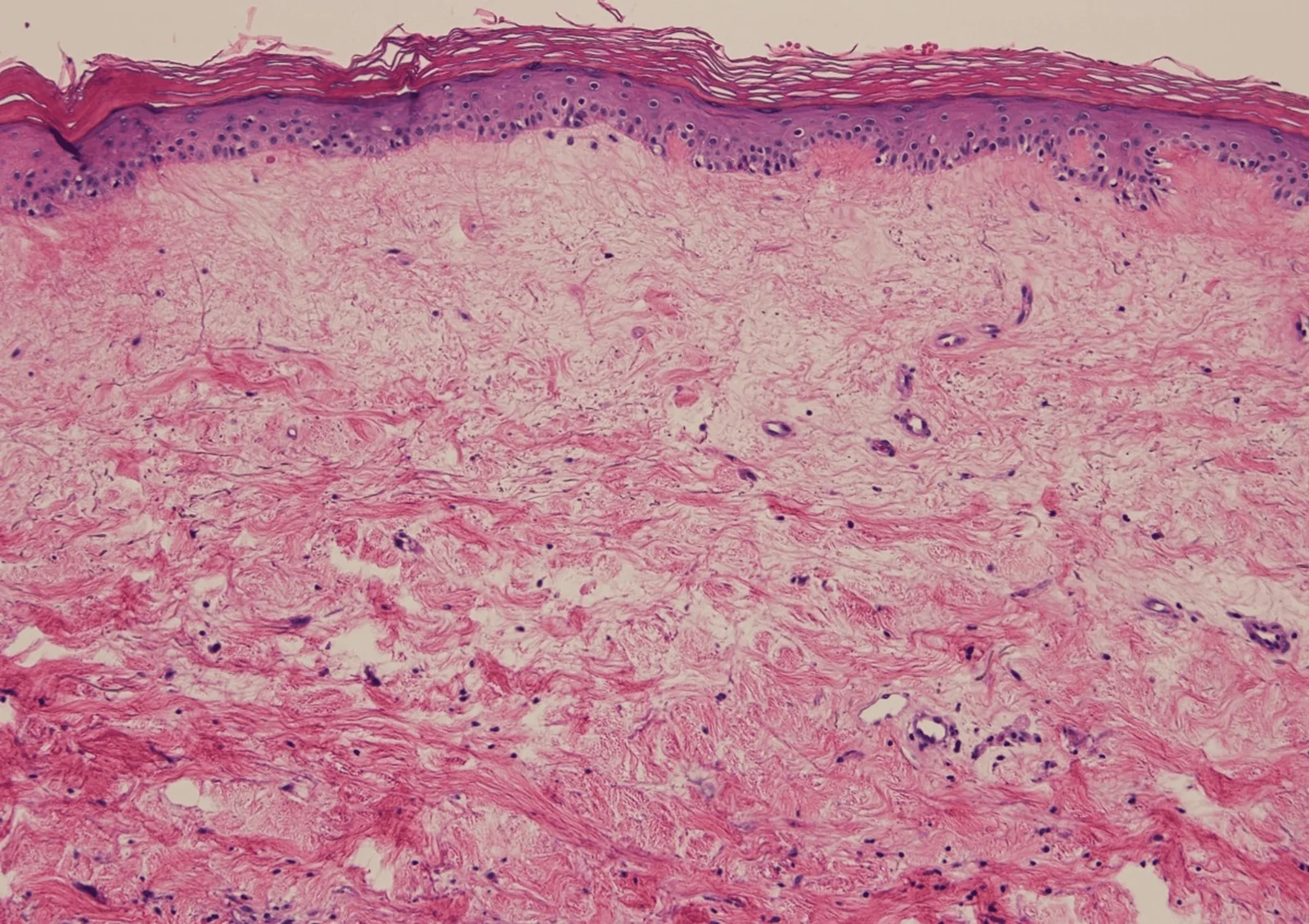
Histopathology Histopathology of lichen sclerosus. Punch biopsy from the left shoulder showing features of lichen sclerosus, including superficial dermal hyalinization beneath an attenuated orthokeratotic epidermis. Hematoxylin and eosin stain, original magnification 100x.

The patient experienced severe pruritus associated with impaired sleep and reduced daytime function. For symptom reporting, pruritus intensity was assessed using a patient-reported 11-point numerical rating scale (NRS), with 0 representing no itch and 10 representing the worst imaginable itch; reported scores reflected average itch intensity over the prior week. Her baseline itch score was 8/10. We considered the pruritus refractory because it remained severe and functionally impairing despite a three-month course of betamethasone dipropionate 0.05% cream, six sessions of intralesional triamcinolone acetonide 2.5 mg/mL with only partial relief, and prior office-based narrowband ultraviolet B phototherapy.

In 2021, she underwent office-based narrowband ultraviolet B phototherapy three times weekly for approximately three months. This yielded marked symptomatic improvement with prolonged remission of pruritus and clinical stability until early 2026. The degree of complete lesional clearance after phototherapy was not documented. Four months before nemolizumab initiation, she developed recurrent and worsening pruritus in areas of known truncal lichen sclerosus.

Because of persistent severe itch despite prior topical corticosteroid therapy, intralesional corticosteroid therapy, and prior phototherapy, off-label nemolizumab was started. The patient weighed 64 kg. She received nemolizumab 60 mg subcutaneously as a loading dose, administered as two 30 mg pens, followed by a 30 mg maintenance dose at week four, consistent with the labeled maintenance dosing used in lower-weight adults. No concomitant topical, systemic, antipruritic, or phototherapy treatment was used during the first eight weeks after nemolizumab initiation.

At four-week follow-up, the patient reported complete resolution of pruritus. Her itch NRS improved from 8/10 before nemolizumab to 0/10 at week four. She reported substantial improvement in sleep and daytime function and was asymptomatic from an itch standpoint. Examination continued to show persistent shiny white atrophic plaques involving the upper back, chest, and shoulders, without visible change in the number or appearance of lesions. She reported no adverse effects. A second dose of nemolizumab 30 mg subcutaneously was administered at week four.

At eight-week follow-up, pruritus remained markedly improved, with an itch NRS of 0/10. The patient reported a flare of burning in the perianal area several weeks earlier that had already markedly improved by the time of evaluation. Examination at week eight showed shiny white plaques involving the perianal area, without vulvar involvement. Because a dedicated baseline perianal examination before nemolizumab was not documented, the precise timing of active perianal disease relative to treatment initiation cannot be established with certainty. She reported no adverse effects from nemolizumab, and insurance approval had been obtained. Nemolizumab was continued at 30 mg subcutaneously every four weeks. Because of active perianal disease, clobetasol 0.05% ointment was started once daily to the affected perianal area for up to one week, with instructions to stop for at least one week before any additional short pulse.

Follow-up photography of the same right upper back lesion at week eight showed persistent plaque morphology without visible clinical resolution despite marked symptomatic improvement (Figure 3). The chronic nature of lichen sclerosus and the importance of ongoing anogenital surveillance were reviewed, including prompt evaluation of any persistent erosion, ulceration, bleeding, pain, induration, nodularity, or nonhealing area.

**Figure 3:**
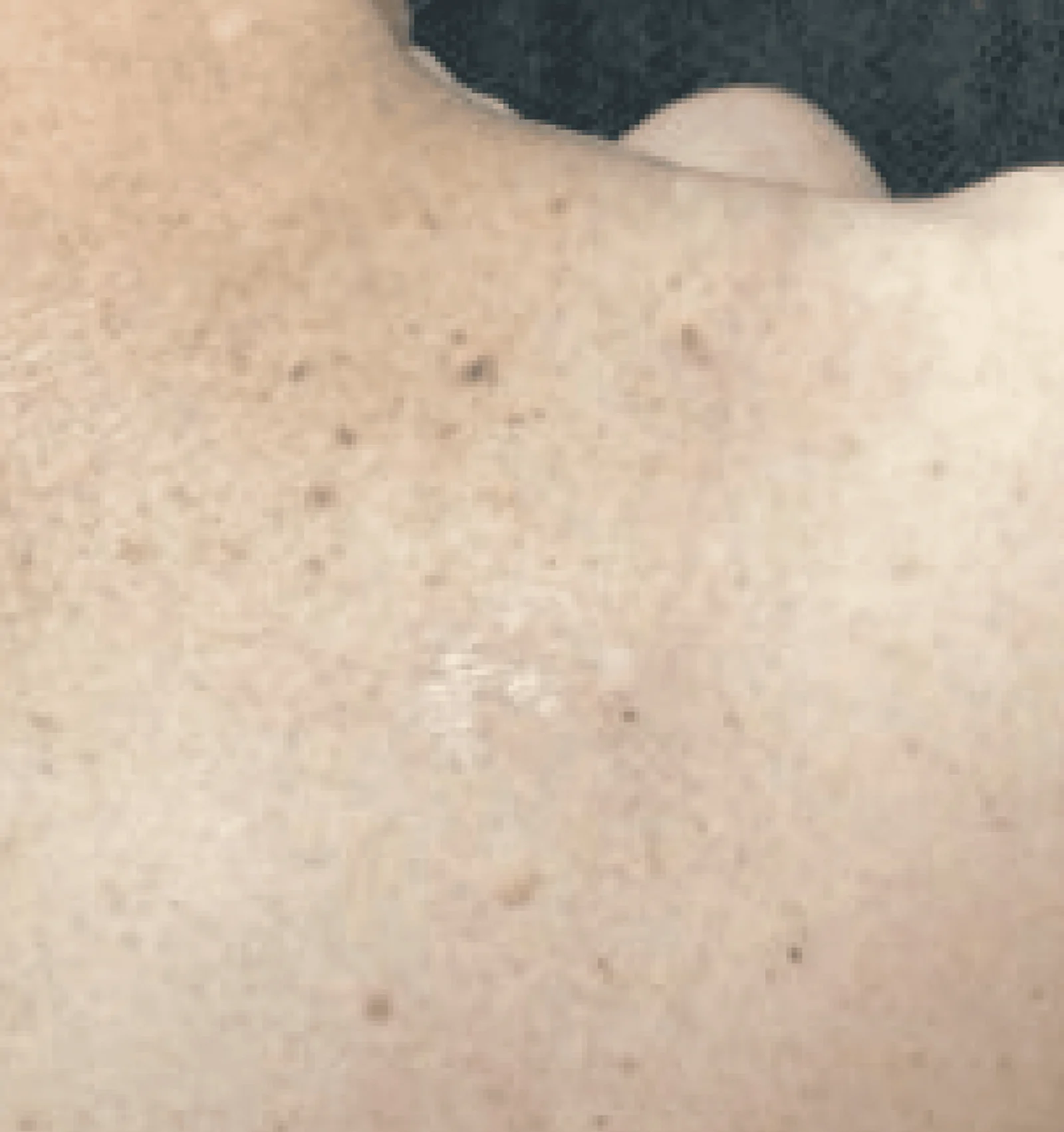
Persistent plaque morphology after eight weeks of nemolizumab Follow-up photograph of the same right upper back lesion after eight weeks of nemolizumab therapy, demonstrating persistent shiny white plaque morphology without visible lesional resolution despite marked improvement in pruritus.

## Discussion

This case describes a temporal association between nemolizumab initiation and rapid, sustained improvement of severe refractory pruritus in biopsy-proven lichen sclerosus. The initial symptomatic response occurred during the first eight weeks of therapy without concomitant topical therapy, systemic therapy, antipruritic therapy, or phototherapy. Importantly, the patient’s truncal plaques did not visibly improve during this interval, and active perianal disease was documented at week eight. Serial clinical photographs of the same truncal lesion further support the dissociation between pruritic burden and visible plaque morphology.

Extragenital lichen sclerosus is less common than anogenital disease and may be underrecognized. It often presents as white or hypopigmented atrophic papules and plaques and may involve the trunk, shoulders, neck, and proximal extremities [2,3]. In this patient, several truncal plaques are localized to scars from prior post-bariatric body-contouring surgery. Lichen sclerosus has been reported in sites of prior cutaneous injury, consistent with koebnerization or an isomorphic response [9,10]. The scar-associated distribution in this case suggests that prior surgical trauma may have served as a localizing factor for the truncal lesions, although it does not fully explain the later recurrence of severe pruritus or the subsequently documented perianal disease.

The patient’s prior response to narrowband ultraviolet B phototherapy is consistent with published reports supporting phototherapy as a therapeutic option in selected patients with extragenital lichen sclerosus [5,6]. In this case, a three-month course of office-based narrowband ultraviolet B yielded prolonged symptomatic remission until early 2026. However, severe pruritus later recurred, again affecting sleep and daytime function.

Nemolizumab targets interleukin-31 receptor alpha, a pathway strongly implicated in chronic itch [7,8]. However, the therapeutic rationale in lichen sclerosus should be interpreted cautiously. Direct mechanistic evidence supporting an interleukin-31-driven pathway in lichen sclerosus remains limited, and the biologic rationale for treatment in this case is extrapolated from other pruritic inflammatory dermatoses. Even so, the patient’s itch NRS improved from 8/10 at baseline to 0/10 at both week four and week eight, despite the absence of visible truncal plaque resolution and the presence of active perianal disease at the latter visit. These findings suggest potential symptomatic benefit from interleukin-31 receptor alpha blockade without establishing disease modification.

This report has several limitations. It describes a single patient with relatively short follow-up. Pruritus improvement was assessed using a nonvalidated patient-reported 0-10 numerical rating scale rather than a validated itch-specific instrument. Baseline perianal examination findings before nemolizumab were not documented, limiting conclusions about the effect of treatment on anogenital disease. In addition, the patient began clobetasol for active perianal disease only after the eight-week assessment, so any subsequent improvement in that area cannot be attributed solely to nemolizumab. Finally, the absence of early visible morphologic improvement limits any inference that nemolizumab alters the underlying sclerosis, atrophy, or long-term disease course of lichen sclerosus.

## Conclusions

In this patient with truncal lichen sclerosus and later documented perianal involvement, off-label nemolizumab was temporally associated with rapid and sustained improvement in severe refractory pruritus during the first eight weeks of treatment. This symptomatic response occurred without early visible resolution of truncal plaque morphology. Although this observation suggests potential antipruritic benefit, it does not establish nemolizumab as a treatment for lichen sclerosus or demonstrate disease modification. Further controlled investigation is needed to clarify the role of interleukin-31 receptor alpha blockade in lichen sclerosus-associated itch.
